# Supplementary material for: Numeracy skills learning of children in Africa:—Are disabled children lagging behind?
Source: PLoS One. 2023 Apr 20;18(4):e0284821. doi: 10.1371/journal.pone.0284821 (PMC10118103; doi:10.1371/journal.pone.0284821)
Supplement: S1 Table — (PDF) [file pone.0284821.s001.pdf]

S1 Table Sample size of children who have done the numeracy test by disability status and country

Country	Non-disabled	Disabled	Vision disabled	Hearing disabled	Physical disabled	Intellectual disabled	Multiple disabled	Total
DRCongo	6,268	395	13	12	108	215	47	6,663
The Gambia	3,104	128	8	3	34	75	8	3,232
Ghana	4,372	542	16	11	58	429	28	4,914
Lesotho	2,567	141	42	19	10	61	9	2,708
Sierra Leone	4,761	324	6	9	126	145	38	5,085
Togo	2,252	202	28	12	16	135	11	2,454
Tunisia	2,135	168	27	5	36	79	21	2,303
Zimbabwe	3,660	235	19	16	13	169	18	3,895
Total	29,119	2,135	159	87	401	1308	180	31,254
