# Supplementary material for: Numeracy skills learning of children in Africa:—Are disabled children lagging behind?
Source: PLoS One. 2023 Apr 20;18(4):e0284821. doi: 10.1371/journal.pone.0284821 (PMC10118103; doi:10.1371/journal.pone.0284821)
Supplement: S2 Table — (PDF) [file pone.0284821.s002.pdf]

S2 Table Regression results for estimating the determinant factors of each disability type

	Vision disabled (1=yes, 0=no)	Hearing disabled (1=yes, 0=no)	Physical disabled (1=yes, 0=no)	Intellect disabled (1=yes, 0=no)	Multiple disabled (1=yes, 0=no)
**Age**	0.000	0.000	-0.003***	-0.001	-0.001
**Gender (1=girl, 0=boy)**	-0.002	-0.001	0.001	-0.009*	-0.001
**Area (1=rural, 0=urban)**	0.001	0.001	0.003	-0.004	0.000
**Gender of household head (1=female, 0=male)**	-0.001	-0.003	0.005	0.001	-0.002
**Highest completed educational level of household head (base category: primary)**					
Primary	0.002	0	0.002	-0.004	0.000
Lower secondary	-0.001	0.000	0.005	-0.004	-0.004*
Upper secondary	0.002	0.003	-0.001	-0.024**	0.006
Higher education	0.002	0.001	-0.008**	-0.011	-0.002
**Family structure (base category: live together with both mother and father)**					
Only mother	0.007	0.003	-0.004	0.003	0.003
Only father	0	0.000	0.005	0.008	-0.001
None of the parents	0.002	0.003	-0.022	0.004	0.008*
**Relationship of the child to the household head (base category: son/ daughter of the household head)**					
Grandchild	-0.005*	0.001	0.017*	0.002	-0.001
Adopted/ foster/ stepchild	-0.005	-0.005***	0.015	0.027	-0.006
Relative	-0.004	-0.002	0.010	0.014	-0.003
Non-relative	-0.009***	0.000	0.018	0.031	-0.009**
**Wealth index (base category: first quintile)**					
Second	0.000	0.000	-0.002	0.001	0.001
Middle	0.002	-0.002	-0.004	-0.006	0
Fourth	0.005	0.000	-0.001	-0.003	-0.001
Highest	0.004	-0.003	0.005	-0.017*	-0.002
**School status of siblings (base category: no sibling)**					
All sblings aged 6-17 currently enrolled in school	-0.003	-0.001	-0.014*	-0.004	0.002
Some siblings 6-17 not currently enrolled in school	0	-0.002	-0.01	-0.017	0
None of sibling currently in school	-0.003	0.000	-0.007	-0.006	0.002
**Number of siblings**	0.001	0.000	0.001	0.002	0
**Country dummy (base category: DRCongo)**					
The Gambia	-0.001	-0.002	-0.006	0.006	-0.003
Ghana	-0.002	0	-0.005	0.074***	-0.002
Lesotho	0.013***	0.004	-0.017***	-0.011	-0.004
Sierra Leone	-0.003	-0.001	0.013*	0.001	0.002
Togo	0.008*	0.003	-0.007	0.033***	-0.003
Tunisia	0.010**	0.001	0.002	0.012	0.003
Zimbabwe	0	0.001	-0.017***	0.019**	-0.005
**Constant**	0.001	0.001	0.051***	0.052**	0.015**
**Sample size**	29722	29648	29974	30905	29796
**R2**	0.006	0.003	0.012	0.02	0.003

Significance levels: * p<0.05; ** p<0.01; *** p<0.001.
