# Supplementary material for: Numeracy skills learning of children in Africa:—Are disabled children lagging behind?
Source: PLoS One. 2023 Apr 20;18(4):e0284821. doi: 10.1371/journal.pone.0284821 (PMC10118103; doi:10.1371/journal.pone.0284821)
Supplement: S3 Table — (PDF) [file pone.0284821.s003.pdf]

S3 Table Sample characteristics

	Non-disabled	Disabled	Vision disabled	Hearing disabled	Physical disabled	Intellectual disabled	Multiple disabled	Total
Numeracy test score	0.57	0.49	0.70	0.60	0.43	0.50	0.34	0.57
Completed school years	3.54	3.04	4.27	3.61	2.40	3.20	2.16	3.50
Age	10.26	10.06	10.46	10.63	9.18	10.28	9.85	10.24
Number of siblings	1.55	1.46	1.45	1.23	1.61	1.43	1.43	1.54
Female (%)	50.6%	48.1%	47.6%	47.9%	50.5%	47.9%	45.6%	50.4%
Rural (%)	62.9%	63.0%	56.5%	75.0%	61.4%	63.3%	64.3%	62.9%
**Family structure (%)**								
Live together with both mother and father	49.4%	46.5%	54.8%	41.8%	52.0%	43.9%	47.7%	49.2%
Only mother	21.8%	22.4%	25.9%	24.2%	21.7%	22.2%	22.0%	21.9%
Only father	6.3%	6.2%	4.8%	5.5%	6.0%	6.6%	5.8%	6.3%
None of the parents	22.5%	24.8%	14.5%	28.6%	20.3%	27.3%	24.5%	22.7%
**Wealth index (%)**								
Poorest	27.4%	28.5%	22.0%	33.3%	29.1%	28.4%	30.3%	27.5%
Second	21.9%	23.0%	16.7%	27.1%	24.2%	22.8%	24.9%	22.0%
Middle	19.9%	20.0%	19.6%	15.6%	16.6%	21.2%	21.6%	19.9%
Fourth	16.2%	16.6%	22.6%	16.7%	16.1%	16.3%	14.9%	16.2%
Richest	14.5%	11.9%	19.0%	7.3%	14.0%	11.3%	8.3%	14.3%
**Highest completed educational level of household head**								
Never in school	32.5%	29.5%	19.2%	32.3%	33.6%	28.9%	31.4%	32.3%
Primary	26.4%	27.8%	35.3%	35.4%	20.7%	28.2%	29.7%	26.5%
Lower secondary	18.8%	22.3%	24.6%	15.6%	16.2%	24.6%	20.9%	19.1%
Upper secondary	16.2%	16.1%	12.0%	11.5%	24.0%	14.8%	14.6%	16.2%
Higher education	6.1%	4.3%	9.0%	5.2%	5.5%	3.5%	3.3%	6.0%
Sample size	30,013	2,293	168	96	422	1,366	241	32,306
