# Supplementary material for: Numeracy skills learning of children in Africa:—Are disabled children lagging behind?
Source: PLoS One. 2023 Apr 20;18(4):e0284821. doi: 10.1371/journal.pone.0284821 (PMC10118103; doi:10.1371/journal.pone.0284821)
Supplement: S4 Table — (PDF) [file pone.0284821.s004.pdf]

S4 Table Regressions on the mean numeracy score with age dummy by countries (non-disabled children)

	DRCongo	The Gambia	Ghana	Lesotho	Sierra Leone	Togo	Tunisia	Zimbabwe
**Age (base category: 7 years old)**								
8	0.081***	0.114***	0.171***	0.168***	0.102***	0.134***	0.067***	0.229***
	(0.011)	(0.022)	(0.022)	(0.025)	(0.015)	(0.024)	(0.017)	(0.022)
9	0.159***	0.263***	0.242***	0.290***	0.207***	0.257***	0.109***	0.380***
	(0.012)	(0.023)	(0.021)	(0.026)	(0.015)	(0.025)	(0.015)	(0.022)
10	0.188***	0.303***	0.325***	0.371***	0.262***	0.275***	0.116***	0.416***
	(0.013)	(0.025)	(0.019)	(0.024)	(0.016)	(0.024)	(0.015)	(0.021)
11	0.265***	0.385***	0.365***	0.388***	0.316***	0.378***	0.138***	0.456***
	(0.013)	(0.026)	(0.019)	(0.025)	(0.017)	(0.023)	(0.016)	(0.021)
12	0.305***	0.482***	0.392***	0.430***	0.361***	0.380***	0.138***	0.514***
	(0.013)	(0.024)	(0.019)	(0.024)	(0.017)	(0.024)	(0.015)	(0.020)
13	0.326***	0.499***	0.421***	0.453***	0.410***	0.434***	0.136***	0.512***
	(0.014)	(0.022)	(0.019)	(0.022)	(0.016)	(0.022)	(0.016)	(0.020)
14	0.361***	0.495***	0.441***	0.445***	0.436***	0.461***	0.144***	0.513***
	(0.013)	(0.024)	(0.019)	(0.024)	(0.019)	(0.022)	(0.015)	(0.020)
**Constant**	0.162***	0.212***	0.408***	0.359***	0.177***	0.362***	0.772***	0.401***
	(0.008)	(0.015)	(0.018)	(0.019)	(0.010)	(0.018)	(0.013)	(0.018)
**Sample size**	6268	3104	4372	2567	4761	2252	2135	3660
**R2**	0.172	0.207	0.191	0.237	0.183	0.223	0.086	0.3

Significance levels: * p<0.05; ** p<0.01; *** p<0.001.
