# Supplementary material for: Numeracy skills learning of children in Africa:—Are disabled children lagging behind?
Source: PLoS One. 2023 Apr 20;18(4):e0284821. doi: 10.1371/journal.pone.0284821 (PMC10118103; doi:10.1371/journal.pone.0284821)
Supplement: S5 Table — (PDF) [file pone.0284821.s005.pdf]

## S5 Table Regression on the completed school years

| | Model 1 | Model 2 | Model 3 | Model 4 | Model 5 |
|---|---|---|---|---|---|
| **Disability status** | | | | | |
| Vision disabled | 0.585*** | 0.153 | 0.539*** | 0.344*** | 0.049 |
| | (0.092) | (0.083) | (0.089) | (0.088) | (0.077) |
| Hearing disabled | -0.201 | -0.438** | -0.111 | -0.152 | -0.326* |
| | (0.172) | (0.152) | (0.174) | (0.179) | (0.156) |
| Physical disabled | -0.325*** | -0.095 | -0.342*** | -0.304*** | -0.134* |
| | (0.070) | (0.066) | (0.066) | (0.065) | (0.061) |
| Intellectual disabled | -0.353*** | -0.429*** | -0.350*** | -0.350*** | -0.395*** |
| | (0.049) | (0.044) | (0.048) | (0.045) | (0.042) |
| Multiple disabled | -1.073*** | -1.078*** | -1.065*** | -1.005*** | -0.995*** |
| | (0.119) | (0.119) | (0.124) | (0.123) | (0.121) |
| **Age (base category: 7 years old)** | | | | | |
| 8 | 0.737*** | 0.723*** | 0.731*** | 0.730*** | 0.714*** |
| | (0.018) | (0.018) | (0.018) | (0.017) | (0.017) |
| 9 | 1.517*** | 1.479*** | 1.485*** | 1.488*** | 1.450*** |
| | (0.022) | (0.021) | (0.022) | (0.021) | (0.021) |
| 10 | 2.214*** | 2.186*** | 2.208*** | 2.212*** | 2.184*** |
| | (0.027) | (0.025) | (0.027) | (0.025) | (0.024) |
| 11 | 3.031*** | 2.962*** | 3.006*** | 3.000*** | 2.941*** |
| | (0.032) | (0.029) | (0.032) | (0.030) | (0.029) |
| 12 | 3.715*** | 3.668*** | 3.699*** | 3.692*** | 3.649*** |
| | (0.037) | (0.034) | (0.036) | (0.035) | (0.034) |
| 13 | 4.513*** | 4.432*** | 4.484*** | 4.464*** | 4.405*** |
| | (0.038) | (0.036) | (0.038) | (0.036) | (0.035) |
| 14 | 5.284*** | 5.192*** | 5.245*** | 5.209*** | 5.143*** |
| | (0.041) | (0.040) | (0.040) | (0.039) | (0.038) |
| **Country dummy (base category: DRCongo)** | | | | | |
| The Gambia | | -0.216*** | | | 0.012 |
| | | (0.054) | | | (0.049) |
| Ghana | | 0.629*** | | | 0.627*** |
| | | (0.048) | | | (0.036) |
| Lesotho | | 1.229*** | | | 1.262*** |
| | | (0.043) | | | (0.039) |
| Sierra Leone | | -0.238*** | | | -0.062 |
| | | (0.047) | | | (0.039) |
| Togo | | 0.652*** | | | 0.736*** |
| | | (0.050) | | | (0.040) |
| Tunisia | | 1.484*** | | | 1.228*** |
| | | (0.040) | | | (0.038) |
| Zimbabwe | | 1.365*** | | | 1.274*** |
| | | (0.038) | | | (0.037) |
| **Area (1=rural, 0=urban)** | | | -0.735*** | | -0.189*** |
| | | | (0.030) | | (0.025) |
| **Gender (1=girl, 0=boy)** | | | | 0.017 | 0.042** |
| | | | | (0.017) | (0.015) |
| **Family structure (base category: live together with both mother and father)** | | | | | |
| Only mother | | | | -0.034 | 0.024 |
| | | | | (0.021) | (0.020) |
| Only father | | | | -0.264*** | -0.117*** |
| | | | | (0.035) | (0.033) |
| None of the parents | | | | -0.425*** | -0.249*** |
| | | | | (0.027) | (0.026) |
| **Number of siblings** | | | | -0.122*** | -0.048*** |
| | | | | (0.007) | (0.007) |
| **Wealth index (base category: first quintile)** | | | | | |
| Second | | | | 0.269*** | 0.287*** |
| | | | | (0.029) | (0.027) |
| Middle | | | | 0.462*** | 0.434*** |
| | | | | (0.030) | (0.027) |
| Fourth | | | | 0.752*** | 0.619*** |
| | | | | (0.032) | (0.033) |
| Highest | | | | 0.987*** | 0.786*** |
| | | | | (0.034) | (0.037) |
| **Highest completed educational level of household head (base category: Primary)** | | | | | |
| Lower secondary | | | | 0.107*** | 0.134*** |
| | | | | (0.023) | (0.021) |
| Upper secondary | | | | -0.060* | 0.309*** |
| | | | | (0.025) | (0.024) |
| Higher education | | | | 0.172*** | 0.333*** |
| | | | | (0.033) | (0.033) |
| Never in school | | | | -0.727*** | -0.291*** |
| | | | | (0.028) | (0.024) |
| **Constant** | 1.097*** | 0.663*** | 1.577*** | 1.218*** | 0.515*** |
| | (0.011) | (0.030) | (0.023) | (0.032) | (0.044) |
| **Sample size** | 32306 | 32306 | 32306 | 31840 | 31840 |
| **R2** | 0.557 | 0.632 | 0.58 | 0.628 | 0.672 |

Significance levels: * p<0.05; ** p<0.01; *** p<0.001.
